# Effectiveness and Safety of a Single-Dose Ivermectin Treatment for Uncomplicated Strongyloidiasis in Immunosuppressed Patients (ImmunoStrong Study): The Study Protocol

**DOI:** 10.3390/pathogens10070812

**Published:** 2021-06-27

**Authors:** Fernando Salvador, Ana Lucas-Dato, Silvia Roure, Marta Arsuaga, Asunción Pérez-Jacoiste, Magdalena García-Rodríguez, José A. Pérez-Molina, Dora Buonfrate, José María Saugar, Israel Molina

**Affiliations:** 1 Infectious Diseases Department, Vall d’Hebron University Hospital, PROSICS Barcelona, 08035 Barcelona, Spain; imolina@vhebron.net; 2 Internal Medicine Department, Hospital Vega Baja, 03314 Orihuela, Spain; helsa_21@hotmail.com; 3 Infectious Diseases Department, North Metropolitan International Health Unit, Germans Trias i Pujol University Hospital, PROSICS North Metropolitan, 08916 Badalona, Spain; sroura.mn.ics@gencat.cat; 4 Tropical Medicine Unit, Infectious Diseases Department, La Paz-Carlos III University Hospital, 28046 Madrid, Spain; martaarsuaga@gmail.com; 5 Internal Medicine Department, Hospital Universitario 12 de Octubre, 28041 Madrid, Spain; mperezja82@hotmail.com; 6 Infectious Diseases Department, Hospital General Universitario de Valencia, 46014 Valencia, Spain; magdala_rod@yahoo.es; 7 National Referral Unit for Tropical Diseases, Infectious Diseases Department, Hospital Ramón y Cajal, IRYCIS, 28034 Madrid, Spain; jose.perezmolina@gmail.com; 8 Department of Infectious Tropical Diseases and Microbiology, IRCCS Sacro Cuore Don Calabria Hospital, Negrar, 37024 Verona, Italy; dora.buonfrate@sacrocuore.it; 9 Parasitology Department, Centro Nacional de Microbiología, Instituto de Salud Carlos III, 28222 Madrid, Spain; jmsaugar@isciii.es

**Keywords:** *Strongyloides stercoralis*, immunosuppression, ivermectin, PCR, population pharmacokinetics

## Abstract

Strongyloidiasis affects an estimated 600 million people worldwide, especially in tropical and subtropical areas. Single-dose ivermectin treatment has shown to be effective among immunocompetent patients with uncomplicated strongyloidiasis. Here, we present the protocol of the ImmunoStrong study, a prospective observational study aiming to evaluate the effectiveness and safety of a single-dose ivermectin for treatment of uncomplicated strongyloidiasis in immunosuppressed patients. The secondary objectives are to assess accuracy of molecular techniques for the follow-up of these patients and to determine the population pharmacokinetics of ivermectin. The information retrieved by this study will cover relevant information gaps in the strongyloidiasis management among immunosuppressed patients.

## 1. Introduction

Human strongyloidiasis caused by the nematode *Strongyloides stercoralis* affects an estimated 600 million people worldwide, especially in tropical and subtropical areas [1]. Until now, scarce efforts have been made to reduce its prevalence. However, the World Health Organization has recently included strongyloidiasis among the targets for 2030 soil-transmitted helminth (STH) control [2]. 

Most of the patients with *S. stercoralis* infection are asymptomatic or present with mild symptoms in the form of gastrointestinal disturbances (abdominal pain, diarrhea, vomiting), respiratory symptoms (cough, dyspnea), and skin lesions (rash, larva currens) [3]. This parasite can persist in the host indefinitely in the absence of reinfections thanks to its ability to autoinfect the human host. Under immunosuppressive conditions such as corticosteroid therapy, solid organ transplantation, or human T lymphotropic virus 1 (HTLV-1) infection, its cycle of autoinfection can be amplified, leading to severe clinical presentations with high mortality, such as *Strongyloides* hyperinfection syndrome and disseminated strongyloidiasis [4,5]. 

Autochthonous cases of trongyloidiasis in Western European countries have been described, although they are currently anecdotal, restricted to specific areas, and mostly affecting elderly males who worked in agriculture in the past [6,7,8]. However, due to migratory flows and the increase in international travels in recent decades, strongyloidiasis is increasingly diagnosed outside endemic areas [9]. A recent review of the literature showed a prevalence of *S. stercoralis* infection ranging from 10% to 20% in immigrant population living in Spain. These data support the active screening of populations coming from endemic areas given the potential risk of serious presentation in situations of immunosuppression [10]. The frequency of this parasitosis and its potentially deleterious consequences has led the European Center for Disease Control to include strongyloidiasis in the routine screening of migrants coming from endemic areas [11].

The current therapeutic arsenal for the treatment of strongyloidiasis is basically limited to two drugs: Ivermectin and albendazole. There are few clinical trials that have evaluated the efficacy of these drugs for the treatment of strongyloidiasis, showing a cure rate of 76–98% for ivermectin and of 38–78% for albendazole. A recent meta-analysis comparing the efficacy of ivermectin versus albendazole showed the superiority of ivermectin, with ivermectin being the treatment of choice for strongyloidiasis [12]. Ivermectin, discovered in 1970 by the Japanese Nobel laureate Satoshi Omura, is one of the most widely used antiparasitic drugs in both human and animals. Its mechanism of action is not yet exactly known, and it has “in vitro” activity against various helminths (*S. stercoralis*, *Trichuris trichiura*, hookworms, different species of filarial worms, *Gnathostoma* sp), ectoparasites (lice, scabies), virus (dengue, the novel SARS-CoV-2), and mosquitoes (of the genus *Anopheles*) [13]. The duration of ivermectin treatment for strongyloidiasis was a matter of debate until a few years ago. However, a recent randomized clinical trial conducted in a nonendemic area (Strong Treat 1 to 4) compared a single dose of ivermectin (200 mcg/kg/day) versus four doses (a dose of 200 mcg/kg/day given for 2 consecutive days, and another two doses given 14 days apart), with a 12-month follow-up using classical parasitological tests, *S. stercoralis* serology, and detection of *S. stercoralis* DNA through polymerase chain reaction (PCR). The results showed that there were no differences between the two regimens (efficacy 86% versus 85% respectively, *p* = 0.75). Furthermore, similar cure rates were observed at 6 and 12 months after treatment [14]. Immunosuppressed patients were not included in this clinical trial. Thus, a lack of evidence about the efficacy of the single-dose regimen remains for this population.

The aim of the ImmunoStrong Study is to evaluate the effectiveness and safety of a single-dose ivermectin for the treatment of uncomplicated strongyloidiasis in immunosuppressed patients.

## 2. Methods

The ImmunoStrong Study is a prospective observational study where adult immunosuppressed (IS) patients with uncomplicated strongyloidiasis will be included. Control group will be composed by immunocompetent (IC) patients with uncomplicated strongyloidiasis. The study will be carried out in 8 sites in Spain and Italy: Vall d’Hebron University Hospital (Barcelona, Spain), Germans Trias I Pujol University Hospital (Badalona, Spain), Ramón y Cajal University Hospital (Madrid, Spain), La Paz-Carlos III University Hospital (Madrid, Spain), 12 de Octubre University Hospital (Madrid, Spain), Vega Baja Hospital (Orihuela, Spain), General University Hospital of Valencia (Valencia, Spain), and IRCCS Ospedale Sacro Cuore-Don Calabria (Negrar, Italia). The Parasitology Reference and Research Laboratory of the National Microbiology Center of the Carlos III Health Institute (ISCIII, Madrid, Spain) will perform the molecular diagnosis techniques.

### 2.1. Patient Eligibility

Inclusion criteria will include: Adult patients (≥18 years old) who sign the informed consent, diagnosis of strongyloidiasis either by direct parasitological methods (positive coproparasitological examination and/or agar plate culture) or by positivity of a serological technique (ELISA) with an optical density (OD) index greater than or equal to 2, and current residence in an area without evidence of active transmission of strongyloidiasis. The exclusion criteria will be as follows: Pregnancy or lactation, intolerance or allergy to ivermectin, renal failure (glomerular filtration rate equal to or less than 30 mL/min) or advanced liver disease (Child-Pugh B or C), coinfection by *Loa loa* (due to the risk of presenting encephalopathy when administering ivermectin), complicated strongyloidiasis (*S. stercoralis* hyperinfection syndrome or disseminated strongyloidiasis), planned trip to an area with evidence of active transmission of strongyloidiasis during the next 6 months (to avoid possible reinfections), and previous treatment with ivermectin (in the last year before inclusion).

Patients included in the IS group will be defined by presence of any of the following conditions at enrollment: Treatment with corticosteroids at a cumulative dose greater than or equal to 20 mg/day of prednisone (or equivalent) for at least 10 days, treatment with cytotoxics drugs or antimetabolites, bone marrow or solid organ transplantation, biological drugs, active solid or hematological neoplasia, HIV infection with CD4 lymphocyte count below 200 cells/mm^3^. On the other hand, patients included in the IC group must not have any of the previous conditions (and no HIV infection regardless of the CD4 lymphocyte count).

### 2.2. Outcomes

The primary outcome will be noninferiority of the cure rate of immunosuppressed patients compared to immunocompetent patients. Cure rate is defined as the proportion of patients with a negative direct parasitological test and negative *S. stercoralis* serology or decrease of at least 50% of OD index at 6 months after treatment administration. We used a noninferiority margin of 14% to assess the primary outcome (i.e., lower bound of the 1-sided 95% CI of the difference between cure rates in immunosuppressed versus immunocompetent patients was no more than –14%). The secondary objective will be to compare the safety and tolerability of single dose ivermectin for the treatment of uncomplicated strongyloidiasis between immunosuppressed and immunocompetent patients. Three sub-studies have been prespecified:

1. Evaluation of the usefulness of *S. stercoralis* PCR in feces in the follow-up after treatment of uncomplicated strongyloidiasis in adult patients (proportion of patients with negative *S. stercoralis* PCR after treatment compared to proportion of patients that fulfill the cure criteria),
2. Determination of the population pharmacokinetics (PK) of ivermectin in a single dose for the treatment of uncomplicated strongyloidiasis in adult patients, and
3. Creation of a repository of serum and stool samples from adult patients (immunosuppressed and immunocompetent) with uncomplicated strongyloidiasis (samples before and after treatment with ivermectin) for later use in the evaluation of new diagnostic tools or biomarkers.

### 2.3. Study Protocol

Patients who meet the inclusion criteria will be invited to participate in the study and will be asked to sign the informed consent. All patients must have a coproparasitological study, a *Strongyloides* specific culture, and a *S. stercoralis* serology performed at baseline. The initial evaluation will include: Full blood cell count, basic biochemistry (glucose, kidney function, and liver profile), HIV serology, and detection of microfilaria in peripheral blood to screen for loiasis in patients coming from endemic areas. In addition, a serum sample (for sample repository), a fresh stool sample (for sample repository and *S. stercoralis* PCR), and a plasma sample (for pre-dose ivermectin population PK) will be collected and stored at −80 °C. At the time of inclusion, the time of post-dose peripheral blood (plasma sample) extraction for the ivermectin population PK will be randomly assigned to 8 different points: 1.5, 3, 4, 5, 6.5, 24, 48, and 72 h after treatment (see Table 1).

Patients will be treated with 200 mcg/kg ivermectin (3 mg tablets, Exeltis Laboratory, Madrid, Spain) in a single dose: <50 kg (9 mg), 50–64.9 kg (12 mg), 65–79.9 kg (15 mg), 80–94.9 kg (18 mg), >95 kg (21 mg). Treatment will be directly observed at the hospital or by videocall. Forty-eight hours after the administration of the drug, investigators attempt make a phone call to inquire about possible adverse events using a structured questionnaire. In case of presenting relevant adverse events, the patient could be referred for clinical and analytical evaluation (at treating physician discretion). Follow-up visits will take place 3 and 6 months after treatment and will include the following assessments: Hemogram, *S. stercoralis* serology, coproparasitological study and *Strongyloides* specific culture (in case they were positive at baseline). In addition, a serum sample (for sample repository) and a fresh stool sample (for sample repository and *S. stercoralis* PCR determination) will be collected and stored at −80 °C at both visits. 

### 2.4. Microbiological and Immunological Techniques

Coproparasitological studies, agar plate culture, and *S. stercoralis* serology will be performed in each participant site using their routine techniques. Stool microscopic examination will be performed using one of the following concentration techniques: The Ritchie’s formalin-ether technique, the commercial device Midi and Mini Parasep^®^ (APACOR Ltd., Wokingham, UK), or the commercial device REAL Mini System^®^ (Durviz SL, Paterna, Spain). The specific fecal culture for *S. stercoralis* larvae will be performed using the agar plate or charcoal techniques. Detection of serum anti-*S. stercoralis* IgG will be performed through different enzyme-linked immunosorbent assay (ELISA)-based commercial kits depending on the center: SciMedx *Strongyloides* serology microwell ELISA (SciMedx Corporation, Denville, NJ, USA), NovaLisa *Strongyloides* (NovaTec Immunodiagnostica GmbH, Dietzenbach, Germany), *Strongyloides* IgG IVD-ELISA (DRG Instruments GmbH, Marburg, Germany), ELISA from Bordier (*Strongyloides ratti*; Bordier Affinity Products SA, Crissier, Switzerland), Euroimmun anti-*Strongyloides* ELISA IgG (*Strongyloides papillosus;* Euroimmun AG, Luebeck, Germany). 

Molecular detection of S. stercoralis will be performed at the Parasitology Reference and Research Laboratory of the National Microbiology Center of the Carlos III Health Institute. Genus-specific primers targeting the small subunit ribosomal RNA (SSU rRNA) gene of *Strongyloides* spp. will be used to detect the presence of the parasite in a qualitative real-time PCR (qPCR) assay using SybrGreen reagents (Invitrogen, San Diego CA, USA) as described elsewhere [15].

### 2.5. Ivermectin Population Pharmacokinetics

The technique for quantifying ivermectin concentrations in plasma will be performed by liquid chromatography coupled to electrospray ionization mass spectrophotometry. In this case, a simple liquid-liquid extraction will be made with deuterated ivermectin as an internal standard to normalize the variations in the processing method, and to detect free ivermectin in plasma in a specific way and quantify its concentrations using a standard line. To ensure that it complies with the requirements established by the EMA and FDA for bio-analytical methods, a validation of the ivermectin processing and determination methods will be carried out using an Acquity chromatography system coupled to a Xevo-TQ massas detector (Waters^®^, Milford, MA, USA). Finally, to date, the long-term stability studies for ivermectin detection in plasma samples that have been carried out have shown a stability of 12 months at −20 °C [16]. For this study, we plan to collect the samples, store them, and analyze them within 24 months. For this reason, together with the validation, a long-term stability study will be carried out to cover the range of 12 to 24 months.

### 2.6. Sample Size Calculation and Statistical Analysis

Assuming a response rate of 85% in the immunocompetent group, a 1-sided α value of 5%, a power of 80%, and a maximum of 10% non-evaluable subjects to show noninferiority at a margin of 14%, we estimated the sample size at 180 assessable subjects per group (90 patients in the IS group and 90 patients in the IC group). Inclusion will be performed competitively among all centers.

Categorical data will be presented as absolute numbers and proportions, and continuous variables will be expressed as means and standard deviations (when normal distribution is demonstrated using the Kolmogorov-Smirnov test) or as medians and interquartile ranges. The χ^2^ test or Fisher exact test, when appropriate, will be used to compare the distribution of categorical variables, and the Student’s *t* test or Mann–Whitney U test will be used for continuous variables. Results will be considered statistically significant if the *p* value is <0.05. SPSS software for Windows (Version 19.0; SPSS Inc, Chicago, IL, USA) will be used for statistical analyses. 

### 2.7. Ethical Issues

Procedures will be performed in accordance with the ethical standards described in the Declaration of Helsinki as revised in 2013, and the study protocol has been approved by national regulatory agencies and the Ethical Review Board of the Vall d’Hebron University Hospital with the reference number EOM(AG)008/2021(5790). Written informed consent will be obtained from all participants.

## 3. Discussion

Human strongyloidiasis is still a challenging disease not only in endemic countries, but also in nonendemic countries due to the migration fluxes. Ivermectin has proved to be an effective drug in eliminating the infection, but easier treatment schemes are needed to optimize control strategies and diagnose as much of the infected population as possible [2]. In this regard, the Strong Treat clinical trial has shown high cure rates with a single-dose ivermectin treatment in immunocompetent patients with uncomplicated strongyloidiasis [14]. The ImmunoStrong study aims to continue evaluating the efficacy of a single-dose ivermectin strategy for uncomplicated strongyloidiasis, placing the focus on immunosuppressed patients. 

Information about efficacy of ivermectin for strongyloidiasis in immunosuppressed patients is scarce, since most of the clinical trials have been carried out in immunocompetent patients [12]. Information about treatment efficacy in immunosuppressed patients comes from case series, where patients are usually treated with similar treatment schemes as immunocompetent patients unless they present a severe clinical form. In the absence of disseminated strongyloidiasis or *S. stercoralis* hyperinfection syndrome, efficacy seems to be the same in both groups [17]. The ImmunoStrong study will raise relevant information to optimize treatment schemes in immunosuppressed patients.

The single-dose ivermectin scheme for the treatment of strongyloidiasis opens a range of possibilities and opportunities for the strongyloidiasis control strategies. If this treatment scheme demonstrates efficacy in both groups (immunocompetent and immunosuppressed), it would facilitate mass drug administration campaigns in highly endemic areas. This strategy is already being evaluated by the WHO in pilot studies [2]. A recent study evaluating different strategies of imported strongyloidiasis screening and treatment showed that presumptively treating all immunosuppressed migrants from endemic areas is the most cost-effective strategy [18]. If the efficacy of a single-dose ivermectin scheme is demonstrated in this population, this strategy will become even more affordable. 

The strongyloidiasis diagnosis and follow-up in the ImmunoStrong study is mainly based on serological techniques, which is closer to real-life conditions. Some concerns could arise regarding the sensitivity of serological techniques in immunosuppressed patients, and this fact could affect the patients’ recruitment of the IS group. Regarding specificity issues of the serological techniques, it has been suggested that increasing the cut-off value for positive serology may increase the specificity of the serological test in the diagnosis of confirmed strongyloidiasis [19]. For this reason, patients included in the ImmunoStrong study must have an OD index equal to or greater than 2 in the ELISA test at the time of diagnosis in the absence of larvae detection in parasitological techniques. 

Novel molecular-based diagnostic techniques for strongyloidiasis diagnosis have been developed in the last decades to achieve the highest sensitivity and specificity. A recent meta-analysis, which included 14 studies, evaluated the accuracy of molecular biology techniques for the diagnosis of *S. stercoralis* infection, showing very high specificity of the PCR technique. However, sensitivity showed unsatisfactory results regardless of the reference test used [20]. Moreover, among the 14 included studies, only 1 was performed in immunosuppressed patients, showing scarce benefits of PCR techniques compared with classical parasitological techniques in patients with oncologic diseases [21]. This gap of information will also be covered by the ImmunoStrong study, which will compare the PCR technique with classical parasitological techniques and serology for the strongyloidiasis diagnosis and follow-up after treatment, both in immunocompetent and immunosuppressed patients.

Finally, the present study will perform an ivermectin population pharmacokinetics among the included patients. The information of this sub-study among immunosuppressed patients could be crucial, since this population is usually under a broad range of treatments that could affect the ivermectin concentrations in blood, subsequently affecting the potential efficacy of the single-dose strategy. 

Concluding, the ImmunoStrong study will focus on the strongyloidiasis treatment among immunosuppressed patients, aiming to find an effective and easier method to implement the treatment scheme based on ivermectin. Information about the accuracy of the PCR technique for the diagnosis and follow-up of these patients and the ivermectin population pharmacokinetics will also fill information gaps in this kind of population.

## Figures and Tables

**Table 1:** Visit schedule.

	Screening Period (from −30 Days to Day 0)	Day 0	Population PK Day	Day 2 (Telephone Visit)	Month 3	Month 6
Informed consent	X					
Demographic and clinical information	X					
Concomitant medication	X	X	X	X		
Directed anamnesis	X	X	X	X	X	X
Physical examination	X	X	X		X	X
Ivermectin treatment		X				
Adverse events		X		X		
Full blood cell count	X			X ^a^	X	X
Biochemistry	X			X ^a^		
HIV serology	X					
*Loa loa* screening	X ^b^					
Coproparasitological study	X				X ^c^	X ^c^
Agar plate culture	X				X ^c^	X ^c^
*S. stercoralis* serology	X				X	X
*S. stercoralis* PCR	X				X	X
Serum and stool samples for repository		X			X	X
Plasma sample for PK		X	X			

^a^ In case of presenting relevant adverse events (at treating physician discretion). ^b^ In patients coming from endemic areas for *Loa loa*. ^c^ Only when they were positive at baseline.

## Data Availability

No new data were created or analyzed in this study. Data sharing is not applicable to this article.
